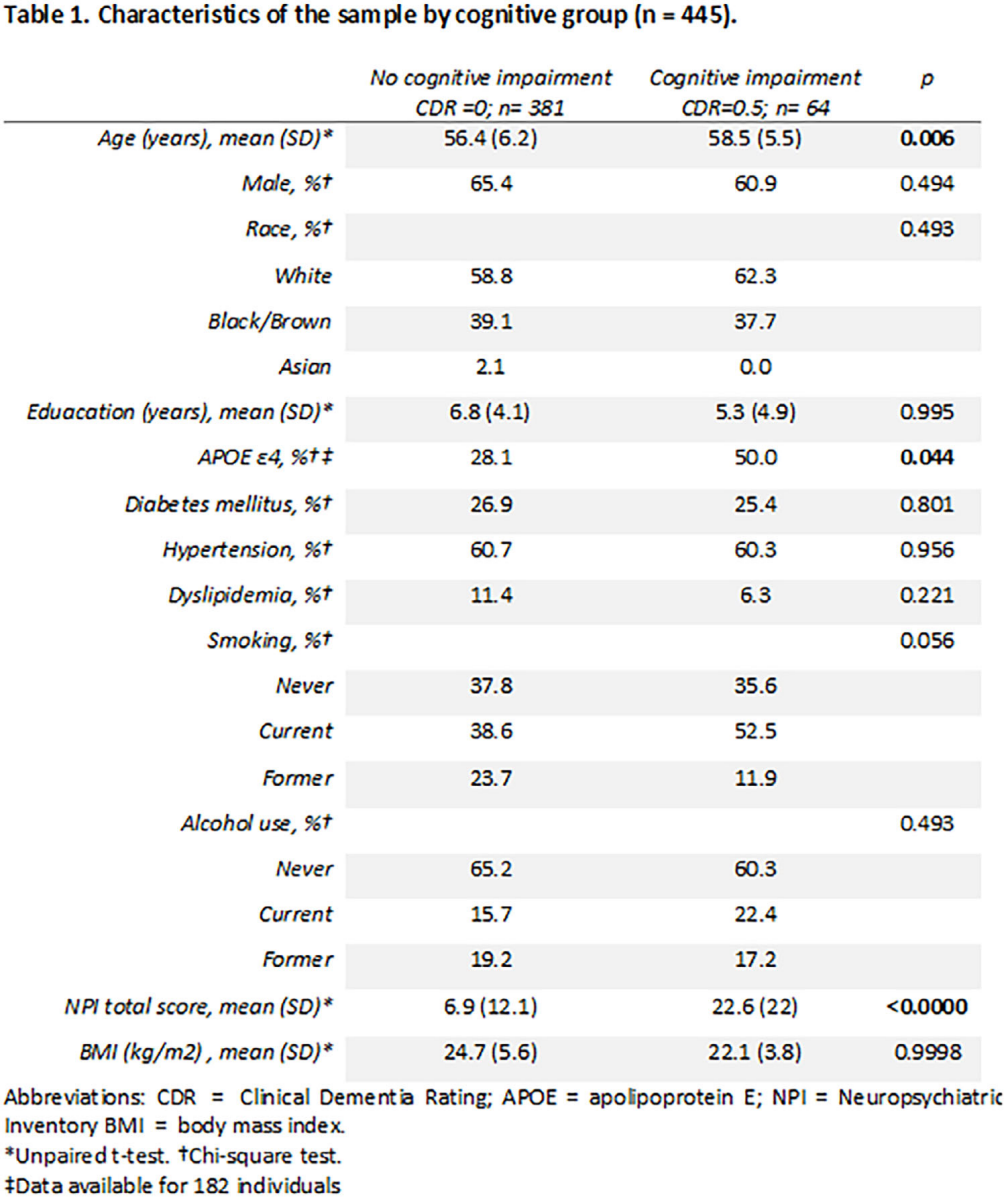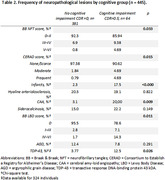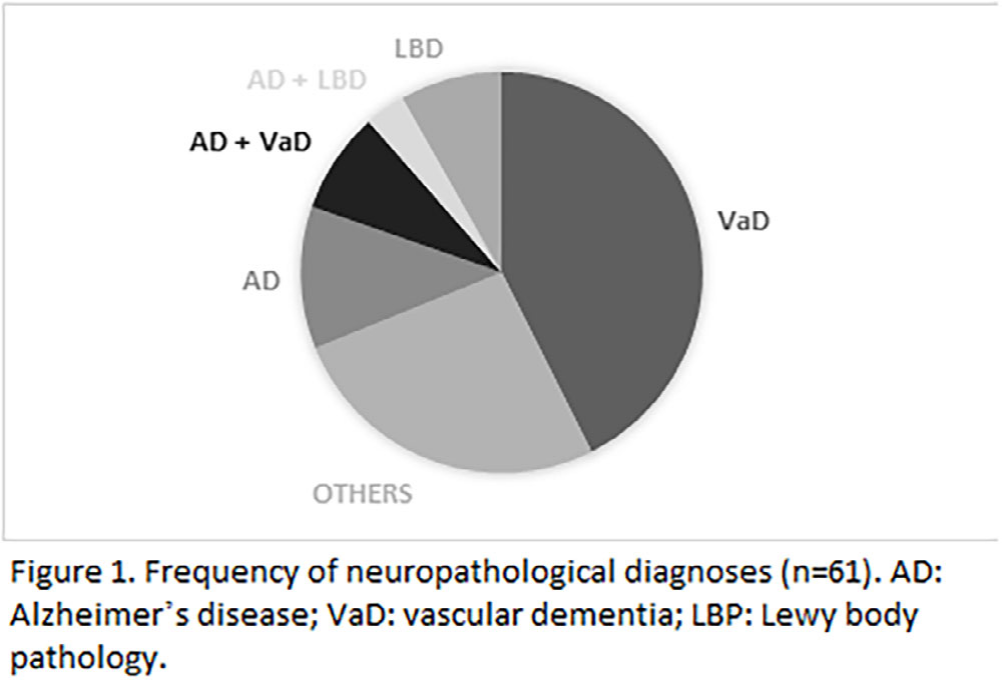# Vascular pathology: a key neuropathological feature in young and middle age individuals

**DOI:** 10.1002/alz.093400

**Published:** 2025-01-03

**Authors:** Renata Elaine Paraizo Leite, Karen L R Socher, Roberta Diehl Rodriguez, Sonia Brucki, Renata Eloah de Lucena Ferretti‐Rebustini, Eduardo Ferriolli, Wilson Jacob‐Filho, Carlos Augusto Pasqualucci, Lea T. Grinberg, Claudia Kimie Suemoto, Ricardo Nitrini

**Affiliations:** ^1^ University of São Paulo Medical School, São Paulo Brazil; ^2^ University of São Paulo Medical School, São Paulo, São Paulo Brazil; ^3^ University of California, San Francisco, San Francisco, CA USA

## Abstract

**Background:**

Clinicopathological studies suggest a role of minor cerebrovascular changes in the cognitive decline of individuals with a low neurodegenerative burden. However, it remains unclear whether small vascular brain lesions can impact cognition in middle aging individuals. Additionally, recent clinicopathological studies have shown that even a low Alzheimer’s disease (AD) neuropathological burden can significantly impact neuropsychiatric function. This study aims to evaluate the presence of AD neuropathological changes and their relationship with cognitive impairment, neuropsychiatric symptoms and risk factors in a multiethnic group under the age of 65.

**Method:**

A post‐mortem study evaluating individuals aged 30‐64 years from the Biobank for Aging Studies at the University of Sao Paulo, Brazil. Neuropathological examinations were carried out based on accepted criteria, using immunohistochemistry. The clinical diagnosis was established through a postmortem interview with an informant using the Cognitive Dementia Rating (CDR). We compared the frequency of neurofibrillary tangles, neuritic plaques, lacunar infarct, hyaline arteriolosclerosis, cerebral amyloid angiopathy, synucleinopathy, and siderocalcinosis between groups. We also described the frequency of neuropathological diagnoses and neuropsychiatric symptoms.

**Result:**

Among participants with neuropathological disease (n = 61), vascular dementia (VaD) was the most frequent diagnosis (42,7%), followed by AD (11,5%) (Figure 1). A higher frequency of vascular pathology (*p*<0.001) was found in individuals with cognitive impairment (Table 2). Some degree of neurofibrillary pathology was found in 54.2% of the individuals, while neuritic plaques were found in 9.7% of the cases, regardless of the presence of cognitive impairment or neuropsychiatric symptoms. AD‐type pathology burden was higher in individuals with cognitive impairment. The frequency of NPS was higher in CI individuals with significant difference in almost all subitems of the NPI, except in appetite changes. High frequency of cerebrovascular risk factors such as hypertension (60.7%) and smoking (61.1%) was also observed in the entire sample (Table 1).

**Conclusion:**

Our data show that AD neuropathological changes can begin in mid‐age and corroborate findings from other series predominantly involving older Caucasians with high educational attainment, which have demonstrated the role of vascular pathology in cognitive impairment. As vascular risks are preventable, implementing aggressive measures to reduce these factors may impact the prevalence of cognition‐related dysfunction.